# Ku70 and Ku80 participate in LPS-induced pro-inflammatory cytokines production in human macrophages and monocytes

**DOI:** 10.18632/aging.103845

**Published:** 2020-10-27

**Authors:** Hong Sun, Quan Li, Gang Yin, Xi Ding, Jing Xie

**Affiliations:** 1 Department of Stomatology, The First Affiliated Hospital of Wenzhou Medical University, Wenzhou, China; 2 Center of Stomatology, The Second Affiliated Hospital of Soochow University, Suzhou, China; 3 Department of Orthopaedics, Wujin Hospital Affiliated to Jiangsu University and The Wujin Clinical College of Xuzhou Medical University, Changzhou, China

**Keywords:** LPS, NFκB, Ku70, Ku80, macrophages and monocytes

## Abstract

In human macrophages and monocytes, lipopolysaccharide (LPS) induces nuclear factor kappa B (NFκB) activation and pro-inflammatory cytokines production. We tested the possible involvement of Ku70 and Ku80 in the process. In THP-1 macrophages and primary human peripheral blood mononuclear cells (PBMCs), shRNA-induced double knockdown of Ku70 and Ku80 potently inhibited LPS-induced production of pro-inflammatory cytokines (TNF-α, IL-1β and IL-6). Additionally, we developed CRISPR/Cas-9 gene-editing methods to knockout both Ku70 and Ku80 in THP-1 cells and PBMCs. Double knockout (DKO) largely inhibited LPS-induced pro-inflammatory cytokines production. Conversely, in THP-1 cells exogenous overexpression of both Ku70 and Ku80 enhanced the pro-inflammatory cytokines production by LPS. Ku70 and Ku80 co-immunoprecipitated with p65-p52 NFκB complex in the nuclei of LPS-treated THP-1 cells. Significantly, LPS-induced NFκB activation was inhibited by Ku70 plus Ku80 double knockdown or DKO. It was however enhanced with Ku70 and Ku80 overexpression. Together, Ku70 and Ku80 promote LPS-induced NFκB activation and pro-inflammatory response in THP-1 cells and human PBMCs.

## INTRODUCTION

Lipopolysaccharide (LPS) is one common pathogen-associated molecular pattern (PAMP) [1, 2]. It is essential in initiation and progression of periodontitis and many other inflammatory diseases [3–5]. LPS is sensed by CD14 and LPS-binding protein (LBP), then binding to its receptor Toll-like receptor 4 (TLR4) on the plasma membrane of macrophages and monocytes [6, 7]. LPS-TLR4 binding will recruit multiple key adaptor proteins, including myeloid differentiation primary response gene 88 (MyD88) and TNF receptor associated factor 6 (TRAF6). This will lead to activation of downstream signaling cascades [6, 7]. Activation of these signalings, including nuclear factor κB (NFκB) and MAP kinase (MAPK) [8, 9], will promote transcription, expression and production of multiple pro-inflammatory cytokines. Several key pro-inflammatory cytokines include tumor necrosis factor-α (TNF-α), interleukin (IL)-1β and IL-6, among others [1, 2]. Our group has been dedicated to understanding the molecular mechanisms. We found that insulin-like growth factor 2 mRNA-binding protein 1 (IGF2BP1) is important for LPS-induced NFκB activation and pro-inflammatory cytokines production [10].

The heterodimeric protein Ku is composed of two subunits: Ku70 and Ku80. The two were originally identified as possible auto-antigens associated with multiple autoimmune diseases, including systemic lupus erythematosus (SLE), scleroderma, polymyositis, and possible others [11, 12]. Ku70 and Ku80 are abundant in eucaryote cells. Both are ubiquitously expressed in cell nuclei [11, 12]. The two recognize and bind ends of DNA double-strand break (DSB), essential for non-homologous end-joining (NHEJ) repair [13–15]. Three structural domains for Ku70/Ku80 proteins have been identified, including the N-terminal domain, the DNA binding domain and the C-terminal domain [16–18]. In the present study, we showed that Ku70 and Ku80 together promoted LPS-induced NFκB activation and pro-inflammatory response in monocytes and macrophages.

## RESULTS

### In human macrophages Ku70 plus Ku80 double knockdown inhibits LPS-induced production of pro-inflammatory cytokines

**Figure 1:**
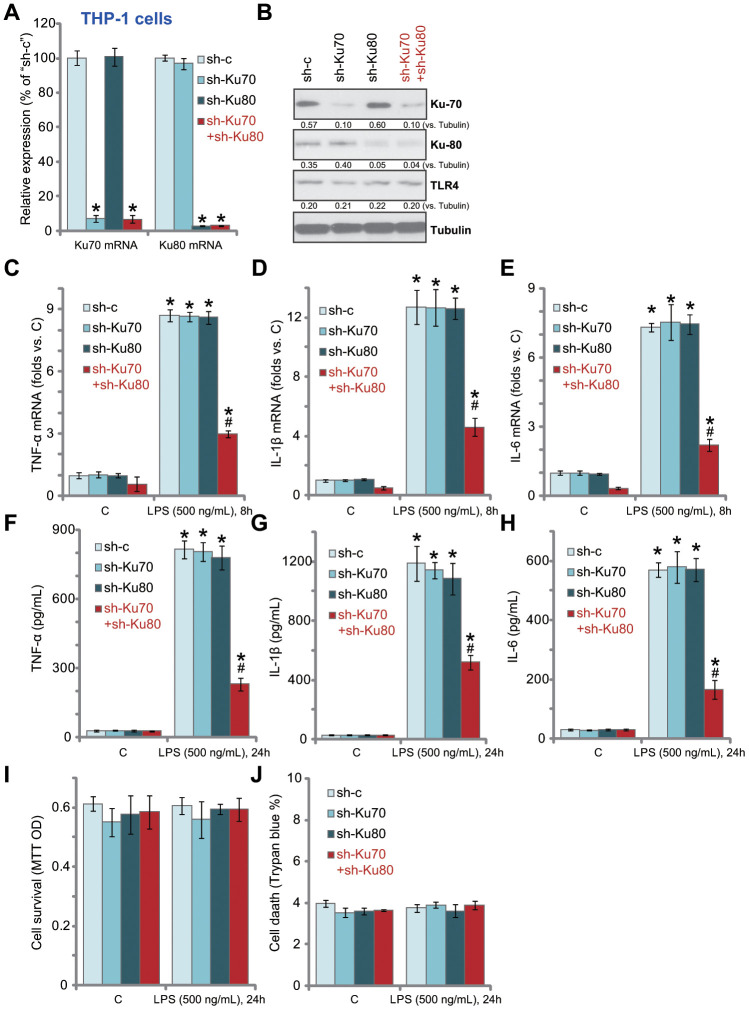
**In human macrophages Ku70 plus Ku80 double knockdown inhibits LPS-induced production of pro-inflammatory cytokines.** THP-1 human macrophages were transduced with Ku70 shRNA lentivirus (“sh-Ku70”) and/or Ku80 shRNA lentivirus (“sh-Ku80”), control cells were treated with scramble control shRNA lentivirus (“sh-c”), stable cells were established following puromycin selection, mRNA and protein expression of listed genes were tested by qPCR (**A**) and Western blotting (**B**); Cells were treated with LPS (500 ng/mL) or vehicle control (“C”) for indicated time, mRNA expression (**C**–**E**) and protein contents in the culture medium (**F**–**H**) of listed pro-inflammatory cytokines (TNF-α, IL-1β and IL-6) were tested by qRT-PCR and ELISA assays; Cell survival and death were tested by MTT (**I**) and Trypan blue staining (**J**), respectively. Expression of listed proteins was quantified, normalized to the loading control (**B**). Data were expressed as mean ± standard deviation (SD, n=5). *p<0.05 *vs.* “C” treatment of “sh-c” cells. ^#^ *p*<0.05. LPS treatment of “sh-c” cells. Experiments in this figure were repeated five times, and similar results were obtained.

In order to study the potential effect of Ku70 and Ku80 in LPS-induced pro-inflammatory response, shRNA strategy was applied. Ku70 shRNA lentivirus and/or Ku80 shRNA lentivirus were transduced to THP-1 human macrophages. Via selection by puromycin stable THP-1 cell lines were established. The qPCR assay results, Figure 1A, confirmed that each of the applied lentiviral shRNA led to dramatic downregulation of target mRNA in THP-1 cells. Cells with both shRNAs presented with significant knockdown of both *Ku70* mRNA and *Ku80* mRNA (Figure 1A). Western blotting analyses of Ku70 and Ku80 protein expression demonstrated similar results as the qPCR results (Figure 1B). TLR4 expression was not altered by knockdown of Ku70 and/or Ku80 (Figure 1B).

Significantly, LPS (500 ng/mL, 8h)-induced mRNA expression of multiple pro-inflammatory cytokines, including *TNF-α* (Figure 1C), *IL-1β* (Figure 1D) and *IL-6* (Figure 1E), was significantly inhibited by Ku70 plus Ku80 double knockdown. While Ku70 or Ku80 single knockdown was completely ineffective (Figure 1C-E). Furthermore, Ku70 plus Ku80 double knockdown in THP-1 cells potently inhibited LPS (500 ng/mL, 24h)-induced TNF-α (Figure 1F), IL-1β (Figure 1G) and IL-6 (Figure 1H) protein section. Ku70 or Ku80 single knockdown again failed to inhibit LPS-induced activity (Figure 1F–1H). In THP-1 cells Ku70 and/or Ku80 knockdown failed to change cell viability (MTT OD, Figure 1I) and death (Trypan blue staining, Figure 1J), even after LPS treatment (Figure 1I and 1J). Thus, Ku70 plus Ku80 double knockdown inhibited LPS-induced production of pro-inflammatory cytokines in THP-1 human macrophages.

### Ku70 plus Ku80 double knockout potently inhibits LPS-induced production of pro-inflammatory cytokines in human macrophages

**Figure 2:**
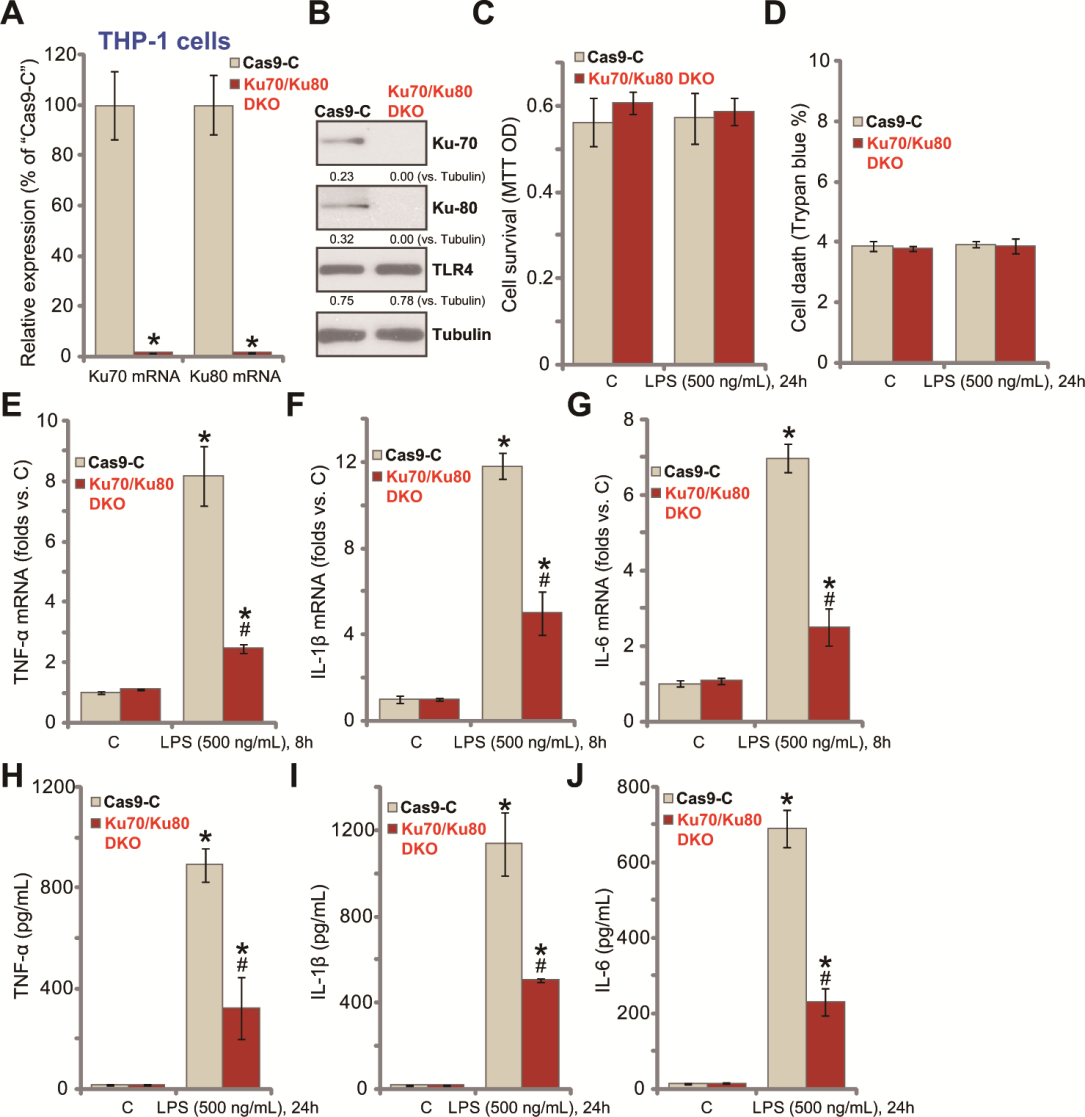
**Ku70 plus Ku80 double knockout potently inhibits LPS-induced production of pro-inflammatory cytokines in human macrophages.** THP-1 human macrophages were transfected with lenti-CRISPR/Cas9-Ku70 KO construct plus lenti-CRISPR/Cas9-Ku80 KO construct, control cells were transfected with CRISPR/Cas9 control vector (“Cas9-C”), stable cells were established following puromycin selection, mRNA and protein expression of listed genes were tested by qPCR (**A**) and Western blotting (**B**); Cells were treated with LPS (500 ng/mL) or vehicle control (“C”) for indicated time, cell viability and death were tested by MTT (**C**) and Trypan blue staining (**D**), respectively; mRNA expression (**E**–**G**) and protein contents in the culture medium (**H**–**J**) of listed pro-inflammatory cytokines (TNF-α, IL-1β and IL-6) were tested by qRT-PCR and ELISA assays; Expression of listed proteins was quantified, normalized to the loading control (**B**). Data were expressed as mean ± standard deviation (SD, n=5). *p<0.05 *vs.* “C” treatment of “Cas9-C” cells. ^#^ *p*<0.05. LPS treatment of “Cas9-C” cells. Experiments in this figure were repeated three times, and similar results were obtained.

In order to exclude the possible off-target effect of the applied Ku70 and Ku80 shRNAs, we established the CRISPR/Cas9-gene editing methods to complete knockout Ku70 and Ku80. As described, the lenti-CRISPR/Cas9-Ku70 KO construct and the lenti-CRISPR/Cas9-Ku80 KO construct were co-transfected to THP-1 human macrophages. Stable cells were established with puromycin selection. As shown, transfection of the two constructs resulted in complete depletion of *Ku70* and *Ku80* mRNA (Figure 2A) and protein (Figure 2B), with TLR4 expression unchanged (Figure 2B). Ku70 and Ku80 double knockout (“Ku70/Ku80 DKO”) failed to inhibit cell viability (Figure 2C) and induce cell death (Figure 2D) in THP-1 cells, regardless of LPS stimulation (Figure 2C and 2D). Importantly, Ku70 plus Ku80 DKO inhibited LPS (500 ng/mL, 8h)-induced mRNA expression of pro-inflammatory cytokines, *TNF-α* (Figure 2E), *IL-1β* (Figure 2F) and *IL-6* (Figure 2G). ELISA assay results demonstrated that TNF-α (Figure 2H), IL-1β (Figure 2I) and IL-6 (Figure 2J) production by LPS (500 ng/mL, 24h) was significantly attenuated with Ku70/Ku80 DKO. These results further support that Ku70 and Ku80 are both important for LPS-induced pro-inflammatory response in THP-1 macrophages.

To achieve single knockout (SKO) cells, THP-1 human macrophages were transduced with the lenti-CRISPR/Cas9-Ku70 KO construct or the lenti-CRISPR/Cas9-Ku80 KO construct. As shown, Ku70 SKO (Supplementary Figure 1A) or Ku80 SKO (Supplementary Figure 1A) did not alter cell viability (Supplementary Figure 1B) nor inducing cell death (Supplementary Figure 1C) in THP-1 cells. LPS-induced production of TNF-α (Supplementary Figure 1D), IL-1β (Supplementary Figure 1E) and IL-6 (Supplementary Figure 1F) was unchanged in THP-1 cells with Ku70 SKO or Ku 80 SKO (*vs.* vector control cells). Therefore, Ku70 or Ku80 SKO failed to inhibit LPS-induced pro-inflammatory response in THP-1 cells.

### Ku70 plus Ku80 double overexpression enhances LPS-induced production of pro-inflammatory cytokines in human macrophages

**Figure 3:**
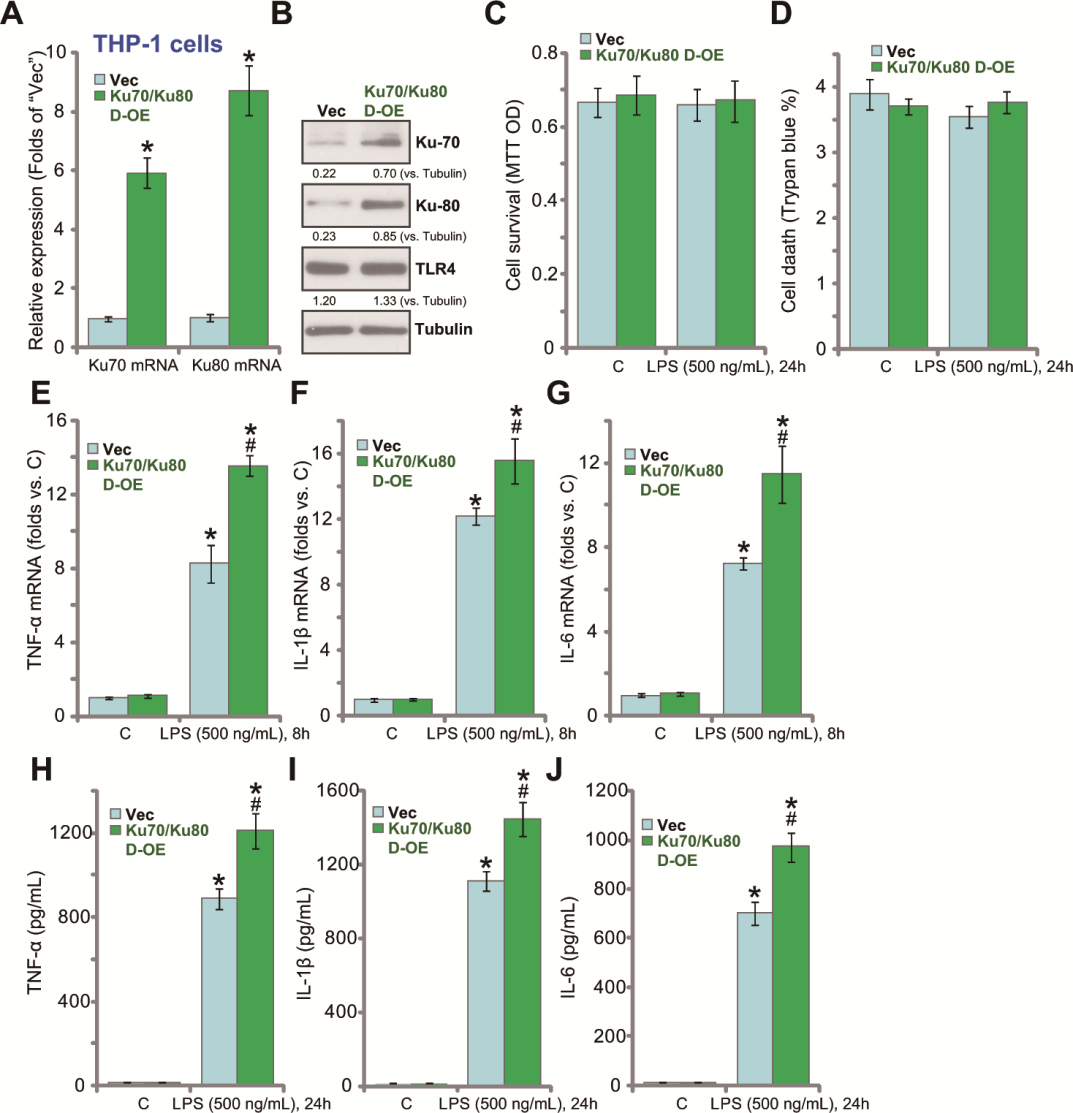
**Ku70 plus Ku80 double overexpression enhances LPS-induced production of pro-inflammatory cytokines in human macrophages.** mRNA and protein expression of listed genes in stable THP-1 human macrophages with the Ku70-expressing AAV plus the Ku80-expressing AAV (“Ku70/Ku80 D-OE”) or with control vector AAV (“Vec”) were shown (**A** and **B**); Cells were treated with LPS (500 ng/mL) or vehicle control (“C”) for indicated time, cell survival and death were tested by MTT (**C**) and Trypan blue staining (**D**), respectively; mRNA expression (**E**–**G**) and protein contents in the culture medium (**H**–**J**) of listed pro-inflammatory cytokines (TNF-α, IL-1β and IL-6) were tested by qRT-PCR and ELISA; Expression of listed proteins was quantified, normalized to the loading control (**B**). Data were expressed as mean ± standard deviation (SD, n=5). *p<0.05 *vs.* “C” treatment of “Vec” cells. ^#^ *p*<0.05. LPS treatment of “Vec” cells. Experiments in this figure were repeated four times, and similar results were obtained.

Based on the results above, Ku70 plus Ku80 overexpression could possibly enhance LPS-induced pro-inflammatory response. Therefore, the Ku70-expressing adeno-associated virus (AAV) plus the Ku80-expressing AAV were co-transfected to THP-1 macrophages. Stable cells were established by puromycin selection. Analyzing mRNA expression, by qPCR, confirmed that *Ku70* mRNA and *Ku80* mRNA levels increased over 6-8 folds in the stable cells (“Ku70/Ku80 D-OE” cells, Figure 3A). Ku70 and Ku80 proteins were upregulated as well in Ku70/Ku80 D-OE cells (Figure 3B). Ku70 plus Ku80 overexpression did not change cell viability (Figure 3C) nor inducing cell death (Figure 3D) in THP-1 cells. Significantly, in the D-OE cells, LPS-induced mRNA expression of pro-inflammatory cytokines, *TNF-α* (Figure 3E), *IL-1β* (Figure 3F) and *IL-6* (Figure 3G), was indeed enhanced. ELISA assays further confirmed that the production of TNF-α (Figure 3H), IL-1β (Figure 3I) and IL-6 (Figure 3J) by LPS was augmented.

In THP-1 cells ectopic overexpression of Ku70 or Ku80 (single overexpression) (Supplementary Figure 2A) did not change cell viability (Supplementary Figure 2B) or induce cell death (Supplementary Figure 2C). Significantly, in Ku70-overxpressed or Ku80- ovexpressed THP-1 cells, LPS-induced production of TNF-α (Supplementary Figure 2D), IL-1β (Supplementary Figure 2E) and IL-6 (Supplementary Figure 2F) was unchanged. Thus, single Ku70 or Ku80 overexpression of failed to promote LPS-induced production of pro-inflammatory cytokines in THP-1 cells.

### Ku70 and Ku80 silencing or KO inhibits LPS-induced production of pro-inflammatory cytokines in human PBMCs

**Figure 4:**
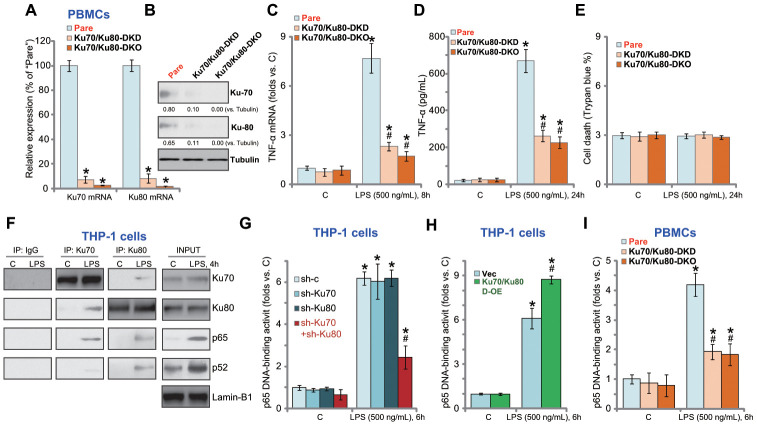
**Ku70 and Ku80 silencing or KO inhibits LPS-induced pro-inflammatory cytokines production and NFκB activation in human PBMCs.** The primary PBMCs were transfected with Ku70 shRNA lentivirus plus Ku80 shRNA lentivirus (“Ku70/Ku80-DKD”), or lenti-CRISPR/Cas9-Ku70 KO construct plus lenti-CRISPR/Cas9-Ku80 KO construct (“Ku70/Ku80-DKO”), expression of listed genes was shown (**A** and **B**); Cells were treated with LPS (500 ng/mL) or vehicle control (“C”) for indicated time, *TNF-α mRNA* expression and protein content in the culture medium were tested by qRT-PCR (**C**) and ELISA (**D**); Cell death was tested by Trypan blue staining assay (**E**); The relative NFκB activity was tested by p65 DNA-binding assay (**I**). THP-1 cells were treated with LPS (500 ng/mL) for 4h, nuclear lysate proteins were subjected to co-immunoprecipitation assay (“IP: Ku70/Ku80”) and Western blotting assay (“INPUT”) (**F**). Stable THP-1 human macrophages, bearing control shRNA lentivirus (“sh-c”), Ku70 shRNA lentivirus (“sh-Ku70”) and/or Ku80 shRNA lentivirus (“sh-Ku80”) (**G**), as well as the Ku70-expressing AAV plus the Ku80-expressing AAV (“Ku70/Ku80 D-OE”) or empty vector (“Vec”) (**H**), were treated with LPS (500 ng/mL) or vehicle control (“C”) for 6h, the relative NFκB activity was tested by p65 DNA-binding assay. Expression of listed proteins was quantified, normalized to the loading control (**B**). Data were expressed as mean ± standard deviation (SD, n=5). “Pare” stands for the parental control PBMCs (**A**–**E**, **I**). *p<0.05 *vs.* “C” treatment. ^#^ *p*<0.05. LPS treatment of control cells. Experiments in this figure were repeated four times, and similar results were obtained.

In the primary human PBMCs, Ku70 shRNA lentivirus and Ku80 shRNA lentivirus were applied to silence Ku70 and Ku80 (“Ku70/Ku80-DKD”, Figure 4A and 4B). Furthermore, the lenti-CRISPR/Cas9-Ku70 KO construct and the lenti-CRISPR/Cas9-Ku80 KO construct were co-transfected to PBMCs, resulting in Ku70 and Ku80 double knockout (“Ku70/Ku80-DKO”, Figure 4A and 4B). LPS-induced *TNF-α* mRNA expression (Figure 4C) and protein production (Figure 4D) were largely inhibited in “Ku70/Ku80-DKD” PBMCs and “Ku70/Ku80-DKO” Trypan blue staining assay results show that Ku70 and Ku80 DKD or DKO failed to induce significant cell death in PBMCs (Figure 4E).

### Ku70 and Ku80 are important for LPS-induced NFκB activation in THP-1 cells and primary human PBMCs

NFκB activation is essential for LPS-induced pro-inflammatory cytokines production in macrophages and monocytes [19, 20]. Performing a co-immunoprecipitation (Co-IP) experiments of nuclear lysates in THP-1 cells, we demonstrated Ku70 and Ku80 co-immunoprecipitated with NFκB proteins p52 and p65 in response to LPS stimulation (Figure 4F). “INPUT” results confirmed p52/p65 protein nuclear translocation with LPS stimulation (Figure 4F). Nuclear Ku70 and Ku80 expression was however unchanged (Figure 4F). The NFκB (p65) DNA-binding activity assay results showed that LPS-induced NFκB activation was largely inhibited by shRNA-mediated double knockdown of Ku70 and Ku80 in THP-1 macrophages (Figure 4G). Whereas Ku70 or Ku80 single knockdown was completely ineffective (Figure 4G).

In Co-IP experiments testing cytosol lysates we show that cytosol Ku70 and Ku80 were unable to bind to p65 regardless of LPS stimulation in THP-1 cells (Supplementary Figure 3A). In THP-1 cells Ku70-Ku80 double shRNA did not inhibit p52-p65 entering cell nuclei in response to LPS (Supplementary Figure 3B). Contrarily, exogenous overexpression of Ku70 plus Ku80 (see Figure 3) augmented LPS-induced NFκB activation in THP-1 cells (Figure 4H). In primary human PBMCs, Ku70 and Ku80 double silencing or double KO similarly inhibited LPS-induced NFκB activation (Figure 4I). Therefore, Ku70 and Ku80, by associating with nuclear p52/p65 proteins, are important for LPS-induced NFκB activation in THP-1 cells and primary PBMCs.

## DISCUSSION

The results of the present study suggested that Ku70 and Ku80 both participated in LPS-induced pro-inflammatory response in THP-1 macrophages and primary human PBMCs. It is possible that Ku70 and Ku80 can compensate each other. shRNA-mediated Ku70 and Ku80 double knockdown (DKD) potently inhibited LPS-induced production of pro-inflammatory cytokines (TNF-α, IL-1β and IL-6). While each single knockdown of Ku70 or Ku80 was complete ineffective. Further, CRISPR/Cas-9-mediated double knockout (DKO) of Ku70 and Ku80 largely inhibited LPS-induced pro-inflammatory cytokines production in THP-1 cells and human PBMCs. While single knockout failed to attenuate LPS-induced actions. On the contrary, in THP-1 cells exogenous overexpression of both Ku70 and Ku80 (D-OE) enhanced pro-inflammatory cytokines production by LPS, with Ku70 or Ku80 single OE completely ineffective. These results convincingly showed that Ku70 and Ku80 are both important in regulating LPS-induced pro-inflammatory response in human macrophages and monocytes.

LPS-TLR4 binding will recruit a number of adaptor proteins in TLR4 signaling pathway, activating the IκB kinase (IKK) complex [21, 22]. The latter will phosphorylate IκB, causing IκB ubiquitination and degradation through the proteasome [19, 20]. The cytosol NFκB (p52)/Rel (p65) complex will then be activated by post-translational modifications [19, 20]. Afterwards, p52-p65 complex translocates to cell nuclei, promoting transcription, expression and production of multiple pro-inflammatory cytokines [19, 20].

One important finding of the current study is that Ku70 and Ku80 are important regulators of LPS-induced NFκB activation. We demonstrated that Ku70 and Ku80 co-immunoprecipitated with p65-p52 NFκB complex in nuclei of LPS-treated THP-1 cells, required for full NFκB activation. In THP-1 human macrophages and primary human PBMCs, Ku70 plus Ku80 double knockdown or DKO potently inhibited LPS-induced NFκB activation. Yet, single shRNA or KO did not alter LPS-induced pro-inflammatory responses. On the contrary, forced overexpression of Ku70 plus Ku80 (D-OE) in THP-1 cells potentiated NFκB activation by LPS. Thus, Ku70 plus Ku80 are key regulators of NFκB activation by LPS.

It has been shown that depleting Ku70/Ku80 could reduce cell viability and induce cytotoxicity in certain human cells [23, 24]. Here we found that in THP-1 and PBMCs cells Ku70 and/or Ku80 silencing or KO did not change cell viability nor inducing cell death. Furthermore, forced overexpression of Ku70 and/or Ku80 did not alter cell viability in these cells. Therefore Ku70/Ku80 appear to have a cell type specific effect. Indeed, Ma et al., found that Ku70 silencing using different shRNAs did not alter cell viability in human pancreatic cancer cells [25].

In murine cells the lack of Ku70 could lead to depleted expression of Ku80 [26]. In human cells, however, silencing of Ku70 may not result in depletion of Ku80 [25, 27]. In the current study we showed that Ku70 silencing (by targeted shRNA) did not alter *Ku80* mRNA and protein expression in THP-1 cells. Neither did Ku80 silencing inhibit Ku70 expression. Furthermore, ectopic overexpression of Ku70 did not alter *Ku80* mRNA expression in THP-1 cells. Follow up studies will be needed to further explore the relationship between expression of Ku70 and Ku80 in human macrophages and monocytes.

## CONCLUSIONS

Together, we conclude that Ku70 and Ku80 promote LPS-induced NFκB activation and pro-inflammatory response possibly by enhancing DNA binding of p52-p65 in THP-1 macrophages and PBMCs.

## MATERIALS AND METHODS

### Chemicals, reagents and antibodies

LPS (L4524), puromycin (MABE341), MTT (3-(4, 5-dimethyl-2-thiazolyl)-2, 5-diphenyl-2H-tetrazolium bromide,8 M212), trypan blue dye (T8154) were purchased from Sigma-Aldrich (St. Louis, Mo). Anti-Ku70 (#4588), Anti-Ku80 (#2180), anti-Toll-like Receptor 4 (TLR4, #14358) and other antibodies were purchased from Cell Signaling Technology (Danvers, MA). Fetal bovine serum (FBS, SH10091), RPMI-1640 (SH30027), DMEM (SH30021) and other cell culture reagents were provided by Hyclone (Shanghai, China). mRNA primers, all sequences and vectors were provided by Shanghai Genechem Co. (Shanghai, China). TRIzol (15596026) and other reagents for the RNA assays were purchased from Thermo-Fisher Invitrogen (Shanghai, China). Enzyme-linked immunosorbent assay (ELISA) kits of human IL-1β (557966), IL-6 (550799) and TNF-α (550610) were purchased from BD Biosciences (San Jose, CA).

### THP-1 cell culture

From the Cell Bank of Shanghai Institute of Biological Science (Shanghai, China) THP-1 human macrophages were purchased. Cells were cultured in RPMI-1640 medium plus 10% FBS.

### Human peripheral blood mononuclear cells (PBMCs) primary culture

As previously described [28, 29], by using the lymphocyte separation medium (Sigma, C-44010) PBMCs were collected from healthy donors (all male, 25-35 years old) with the written-informed consent. PBMCs were cultured in DMEM with 10% FBS and necessary supplements [30]. The protocols of this study were approved by the Ethics Committee of Wenzhou Medical University.

### Quantitative real-time reverse transcriptase polymerase chain reaction (qPCR)

At a density of 1.5 ×10 ^5^ cells/well THP-1 cells or the primary human PBMCs were seeded into 12-well plates. Following the applied LPS treatment (500 ng/mL, 8h), TRIzol reagent was utilized to extract total cellular RNA [31, 32]. For qPCR, we utilized an ABI Prism 7600 Fast Real-Time PCR system with the SYBR Green Real-Time Master Mix kit (A46109, Thermo-Fisher, Shanghai, China). The product melting temperatures were calculated by the melt curve analysis. A 2^−ΔΔCt^ method was utilized for the quantification of target mRNA [33], and normalized to the endogenous reference gene *GAPDH*. mRNA primers were listed in Table 1.

**Table 1:** Primers for qPCR assay.

**Genes**	**Forward sequence (5’-3’)**	**Reverse sequence (5’-3’)**
TNF-α	CTCTTCTGCCTGCTGCACTTTG	ATGGGCTACAGGCTTGTCACTC
IL-6	AGACAGCCACTCACCTCTTCAG	TTCTGCCAGTGCCTCTTTGCTG
IL-1β	CCACAGACCTTCCAGGAGAATG	GTGCAGTTCAGTGATCGTACAGG
GAPDH	GTCTCCTCTGACTTCAACAGCG	ACCACCCTGTTGCTGTAGCCAA
Ku-70	GGTTTCAAGCCGTTGGTACTGC	CTCCAGACACTTGATGAGCAGAG
Ku-80	GTTCTAAAGGTCTTTGCAGCAAGA	AAAAGCCACGCCGACTTGAGGA

### Western blotting

At a density of 3 ×10 ^5^ cells/well THP-1 cells or the primary human PBMCs were seeded into six-well plates. Following the applied LPS treatment, 20-30 μg of protein lysates (from each treatment in each lane) were separated by a 10% SDS-PAGE gel, and transferred to a polyvinylidene difluoride (PVDF) blot. After blocking in 10% non-fat milk in PBST, the blot was incubated with applied primary and secondary antibodies. Enhanced chemiluminescence (ECL) reagents (Amersham, Piscataway, NJ) were applied to visualize the immuno-reactive proteins through autoradiography [34]. The quantification of the protein band was through the NIH ImageJ software.

### MTT assay

Briefly, at a density of 1 ×10 ^4^ cells/well THP-1 human macrophage or PBMCs were plated into 96-well plates. After the applied LPS treatment, MTT dye (5 mg/mL, 20 μL in each well) was added. After incubation for another 2-3h, The optical density (OD) of MTT at 490 nm was recorded.

### Cell death assay

At a density of 1 ×10 ^5^ cells/well THP-1 cells or the primary human PBMCs were seeded into the 12-well plates. After the applied LPS treatment, trypan blue staining was performed to quantify cell death. Trypan blue ratio was recorded [35].

### Enzyme-linked immunosorbent assay (ELISA)

Briefly, at a density of 1 ×10 ^4^ cells/well THP-1 human macrophage or PBMCs were plated into 96-well plates. Following LPS treatment, supernatants were collected and cytokines were determined by using the commercial available ELISA kits.

### shRNA

The verified Ku70 lentiviral shRNA (-a) and Ku80 lentiviral shRNA (-a) were provided by Dr. Xiang at Shanghai Jiao Tong University School of Medicine [25]. At a density of 3 ×10 ^5^ cells/well THP-1 cells or the primary human PBMCs were seeded into the six-well plates. The shRNA lentivirus was added to cultured cells (in polybrene-containing medium) for 24h. Afterwards, cells were cultured in fresh complete medium. Puromycin (5.0 μg/mL) was added to select resistant stable cells for four more passages. Expression of Ku70 and Ku80 was tested by qPCR and Western blotting. The non-sense control lentiviral shRNA (“sh-c”, Santa Cruz Biotech) was transfected to the control cells.

### Exogenous Ku70 and Ku80 overexpression

The full-length human *Ku70 cDNA* and *Ku80 cDNA* were provided by Dr. Xiang at Shanghai Jiao Tong University School of Medicine [25], individually sub-cloned into pSuper-flag-puro construct. The construct and the adeno-associated virus (AAV) package plasmids (Genechem) were co-transfected to HEK293 cells to generate Ku70- or Ku80-expressing AAV [36]. At a density of 3 ×10 ^5^ cells/well THP-1 cells were seeded into six-well plates. The virus was enriched, filtered and added to THP-1 cells. Afterwards, cells were cultured in fresh complete medium. Puromycin (5.0 μg/mL) was added to select resistant stable cells for four more passages. Expression of Ku70 and Ku80 was verified by qPCR and Western blotting. Control cells were transfected with AAV with empty vector.

### Ku70 and Ku80 knockout

The lentiviral CRISPR/Cas-9 PX458-green fluorescent protein (GFP) [34] plasmid with sgRNA targeting *human Ku70* (targeted DNA sequence: *CGAGGGCGATGAAGAAGCAG*) or *human Ku80* (targeted DNA sequence: *GATACTGATCCCCACCAGAA*) and a puromycin selection gene were provided by Shanghai Genechem Co. THP-1 cells or primary human PBMCs were seeded into six-well plates at a density of 3 ×10 ^5^ cells/well. Cells were transfected with the lentiviral Ku70 and/or Ku80 knockout (KO) constructs, and selected by puromycin (5.0 μg/mL, for four passages). To obtain monoclonal cells, GFP-positive cells were further sorted by fluorescence activated cell sorting (FACS), and cultured for another two weeks. In the stable cells Ku70 and Ku80 double knockout (DKO) was verified by qPCR and Western blotting. Control cells were transfected with CRISPR/Cas-9 PX458-GFP control plasmid (“Cas9-C”).

### NFκB (p65) DNA-binding activity

Following the applied treatment, nuclear proteins were extracted. By using a TransAM™ ELISA kit (Active Motif, Carlsbad, CA, 43296) the NFκB (p65) DNA-binding activity was tested. For each treatment, 1.0 μg of nuclear extracts were subjected to the binding of p65 to an immobilized consensus sequence in a 96-well plate (1 ×10**^4^** cells/well). After the colorimetric reaction, OD value was measured in an ELISA reader at the test wavelength of 450 nm.

### Co-Immunoprecipitation (Co-IP) assay of nuclear proteins

As previously described [10], the nuclear protein lysates (500 μg proteins in each treatment treatment) were pre-cleared using protein A/G Sepharose (Sigma, Shanghai, China). The pre-cleared nuclear lysates were incubated with anti-Ku70/anti-Ku80 antibody (Cell Signaling Tech, Shanghai, China) for 12h. Afterwards, the protein A/G Sepharose (30 μL for each treatment, Sigma) was added back to the lysates. Ku70/Ku80 co-immunoprecipitation with NFκB protein complex (p52-p65) was subjected.

### Statistics analysis

Data were expressed as mean ± standard deviation (SD). Statistics analyses were performed by using the SPSS software (SPSS Inc., Chicago, IL), with *p* < 0.05 considered as statistical significant [31]. For comparisons among multiple groups, two-way ANOVA with the Bonferroni post hoc testing was performed. A two-tailed unpaired T test (Excel 2007) was applied to test significance between two treatment groups.

## Supplementary Material

Supplementary Figures
