# Supplementary material for: Ku70 and Ku80 participate in LPS-induced pro-inflammatory cytokines production in human macrophages and monocytes
Source: Aging (Albany NY). 2020 Oct 27;12(20):20432–44. doi: 10.18632/aging.103845 (PMC7655212; doi:10.18632/aging.103845)
Supplement: Supplementary Figures [file aging-12-103845-s001..pdf]

SUPPLEMENTARY FIGURES

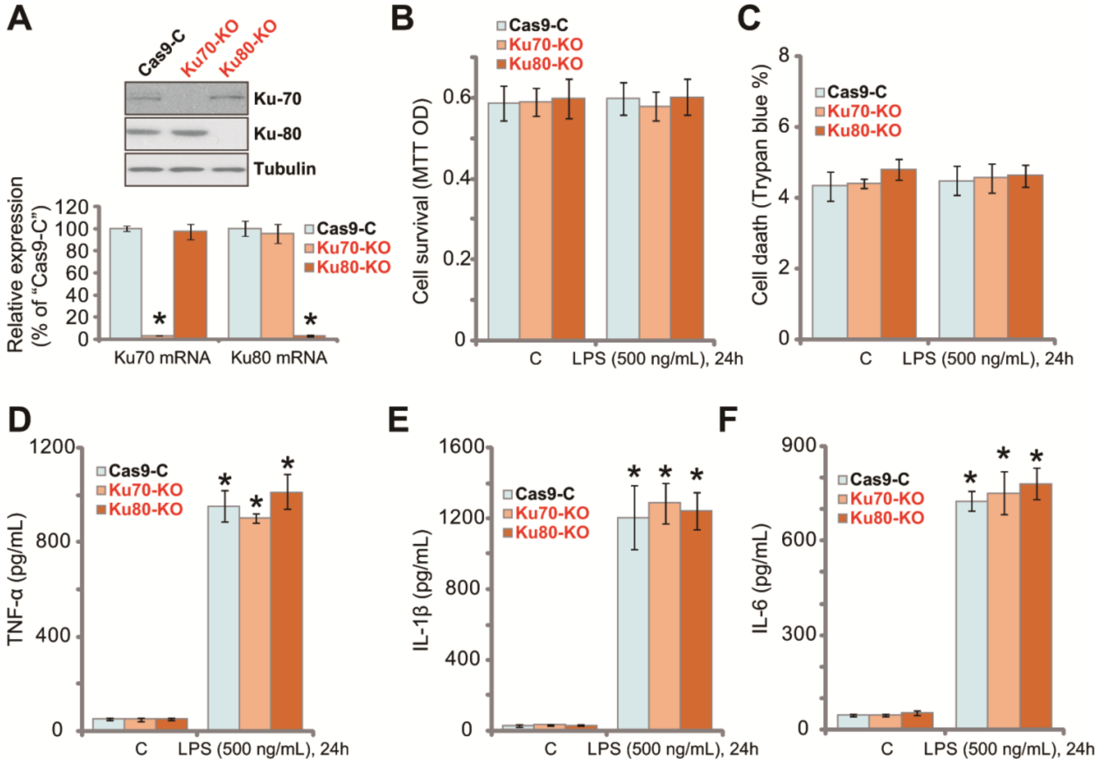

Supplementary Figure 1. THP-1 human macrophages were transfected with lenti-CRISPR/Cas9-Ku70 KO construct ("Ku70-KO") or the lenti-CRISPR/Cas9-Ku80 KO construct ("Ku80-KO"), control cells were transfected with CRISPR/Cas9 control vector ("Cas9-C"); Stable cells were established following puromycin selection, *Ku70* and *Ku80* mRNA and protein expression was tested by qPCR and Western blotting analyses (**A**); Cells were further treated with LPS (500 ng/mL) or vehicle control ("C") for 24h, cell viability and death were tested by MTT (**B**) and Trypan blue staining (**C**), respectively; The cytokines (TNF-α, IL-1β and IL-6) contents in the medium were tested by ELISA assays (**D**–**F**); Data were expressed as mean ± standard deviation (SD, n=5). *p<0.05 *vs.* "C" treatment of "Cas9-C" cells. Experiments in this figure were repeated three times, and similar results were obtained.

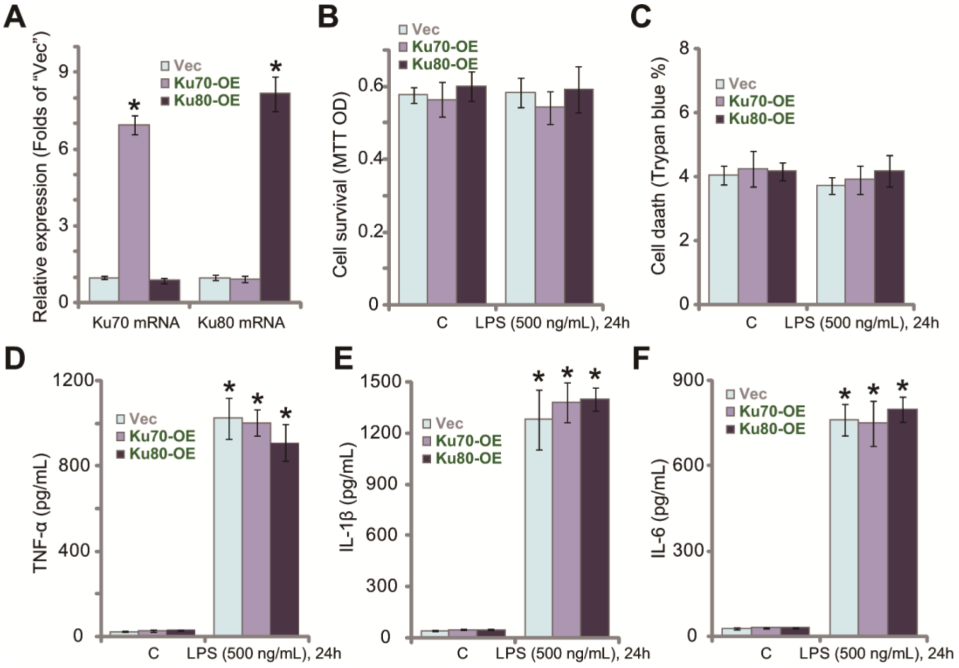

Supplementary Figure 2. *Ku70* and *Ku80* mRNA expression in stable THP-1 human macrophages with the Ku70-expressing AAV construct ("Ku70-OE"), the Ku80-expressing AAV construct ("Ku80-OE") or with control vector AAV ("Vec") was shown (**A**); Cells were treated with LPS (500 ng/mL) or vehicle control ("C") for indicated time, cell viability and death were tested by MTT (**B**) and Trypan blue staining (**C**), respectively; The cytokines (TNF-α, IL-1β and IL-6) contents in the medium were tested by ELISA assays (**D–F**); Data were expressed as mean ± standard deviation (SD, n=5). (**E**) **p*<0.05 *vs.* "C" treatment of "Vec" cells. Experiments in this figure were repeated four times, and similar results were obtained.

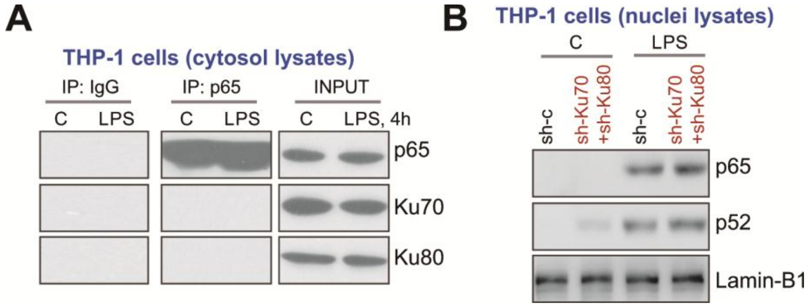

Supplementary Figure 3. THP-1 cells were treated with LPS (500 ng/mL) for 4h, the cytosol lysates were subjected to co-immunoprecipitation assay ("IP: p65") and Western blotting assay ("INPUT") (**A**). Stable THP-1 human macrophages, bearing control shRNA lentivirus ("sh-c"), Ku70 shRNA lentivirus plus Ku80 shRNA lentivirus ("sh-Ku70+sh-Ku80"), were treated with LPS (500 ng/mL) or vehicle control ("C") for 6h, p52-p65 expression in the nuclear lysates was tested (**B**). Experiments in this figure were repeated four times, and similar results were obtained.
